# Unbiased Mutagenesis of MHV68 LANA Reveals a DNA-Binding Domain Required for LANA Function In Vitro and In Vivo

**DOI:** 10.1371/journal.ppat.1002906

**Published:** 2012-09-06

**Authors:** Clinton R. Paden, J. Craig Forrest, Scott A. Tibbetts, Samuel H. Speck

**Affiliations:** 1 Department of Microbiology and Immunology, Emory University School of Medicine, Atlanta, Georgia, United States of America; 2 Immunology and Molecular Pathogenesis Graduate Program, Emory University, Atlanta, Georgia, United States of America; 3 Department of Microbiology and Immunology, University of Arkansas for Medical Sciences, Little Rock, Arkansas, United States of America; 4 Department of Molecular Genetics and Microbiology, University of Florida, Gainesville, Florida, United States of America; University of Southern California Keck School of Medicine, United States of America

## Abstract

The Latency-Associated Nuclear Antigen (LANA), encoded by ORF73, is a conserved gene among the γ2-herpesviruses (rhadinoviruses). The Kaposi's Sarcoma-Associated Herpesvirus (KSHV) LANA is consistently expressed in KSHV-associated malignancies. In the case of the rodent γ2-herpesvirus, murine gammaherpesvirus 68 (MHV68), the LANA homolog (mLANA) is required for efficient virus replication, reactivation from latency and immortalization of murine fetal liver-derived B cells. To gain insights into mLANA function(s), knowing that KSHV LANA binds DNA and can modulate transcription of a variety of promoters, we sought out and identified a mLANA-responsive promoter which maps to the terminal repeat (TR) of MHV68. Notably, mLANA strongly repressed activity from this promoter. We extended these analyses to demonstrate direct, sequence-specific binding of recombinant mLANA to TR DNA by DNase I footprinting. To assess whether the DNA-binding and/or transcription modulating function is important in the known mLANA phenotypes, we generated an unbiased library of mLANA point mutants using error-prone PCR, and screened a large panel of mutants for repression of the mLANA-responsive promoter to identify loss of function mutants. Notably, among the mutant mLANA proteins recovered, many of the mutations are in a predicted EBNA-1-like DNA-binding domain. Consistent with this prediction, those tested displayed loss of DNA binding activity. We engineered six of these mLANA mutants into the MHV68 genome and tested the resulting mutant viruses for: (i) replication fitness; (ii) efficiency of latency establishment; and (iii) reactivation from latency. Interestingly, each of these mLANA-mutant viruses exhibited phenotypes similar to the mLANA-null mutant virus, indicating that DNA-binding is critical for mLANA function.

## Author Summary

The human gammaherpesviruses Epstein-Barr virus (EBV) and Kaposi's sarcoma-associated herpesvirus (KSHV) are tightly associated with a number of different cancers. Unfortunately, due to their very narrow host tropism, characterizing the pathogenesis of these viruses has been difficult. Infection of laboratory mice with the rodent gammaherpesvirus, murine gammaherpesvirus 68 (MHV68), has proven to be an excellent approach for understanding how these viruses cause disease. One of the MHV68 encoded proteins, which is also found in KSHV, is called LANA and in the case of KSHV-associated diseases LANA expression is consistently detected in infected cells. Here we show that the MHV68 LANA shares a key function with the KSHV homolog—namely, modulating gene expression. Using a random mutagenesis protocol, we identified mLANA mutants that had lost transcriptional regulatory activity. We engineered these mutations back into the virus, used the viruses to infect mice, and find that this function is critical to LANA function in vivo and in vitro. This method, combined with the knowledge gained here, sets the stage for future studies to identify mutant forms of LANA that could be used to block wild type LANA function or, alternatively, to design drugs that target LANA function.

## Introduction

Rhadinovirus infections are associated with a number of lymphoproliferative diseases. In the case of the human virus, Kaposi's sarcoma-associated herpesvirus (KSHV, HHV-8), there is tight association between KSHV and Kaposi's sarcoma (sporadic, endemic and HIV-associated forms of Kaposi's sarcoma) [1], as well as multicentric Castleman's disease and primary effusion lymphoma [2], [3]. Herpesvirus saimiri (HVS) has been shown to induce T cell lymphomas [4] and murine gammaherpesvirus-68 (MHV68) has been shown to induce B cell lymphomas [5]. In addition, both HVS and MHV68 can immortalize specific populations of lymphocytes in tissue culture [6], [7]. A common feature of the KSHV-associated malignancies, in addition to harboring the latent viral genome, is the consistent detection of latency-associated nuclear antigen (LANA) expression [8], [9].

LANA, encoded by ORF 73 in the viral genome, is thought to be involved in many aspects of gammaherpesvirus infection. It was discovered as an antigen that speckles the chromosomes of KSHV-infected tumor cells when the tumor cells were stained with KS patient serum [10], [11]. KSHV LANA interacts with a number of cellular proteins that influence cellular signaling events, including interaction with the tumor suppressor p53 [8], [12]–[14]. KSHV LANA has also been shown to bind DNA, which is hypothesized to have importance in loading of replication origins [15]–[18], maintaining the virus genome as an episome [14], [15], [19]–[22] and regulating gene transcription [16], [23], [24]. Domains of the KSHV LANA required for regulating gene transcription and DNA binding have been identified [23], [25], [26].

We have previously shown that the MHV68 LANA (mLANA) is required for replication fitness *in vivo* and *in vitro*, and for virus reactivation from latency [21], [27], [28]. To begin to investigate how mLANA influences virus replication, we have assessed whether mLANA can modulate gene transcription and have identified a promoter in the terminal repeat of MHV68 that is potently repressed by mLANA. We have used this as a tool to identify residues in mLANA that are required for the transcriptional repressor function. Notably, many of the identified mutations that eliminate mLANA-mediated transcriptional repression are within the conserved (among rhadinovirus LANA proteins) C-terminal region of mLANA, corresponding to a putative DNA-binding domain. Investigation of these mutations reveals that the DNA-binding function is indeed disrupted. We further engineered several of these mutations into the MHV68 genome and determined that mLANA mutants deficient in transcriptional repressor function have a profound effect on both virus replication and reactivation from latency.

## Results

### mLANA-mediated repression of transcription from the MHV68 terminal repeat

**Figure 1:**
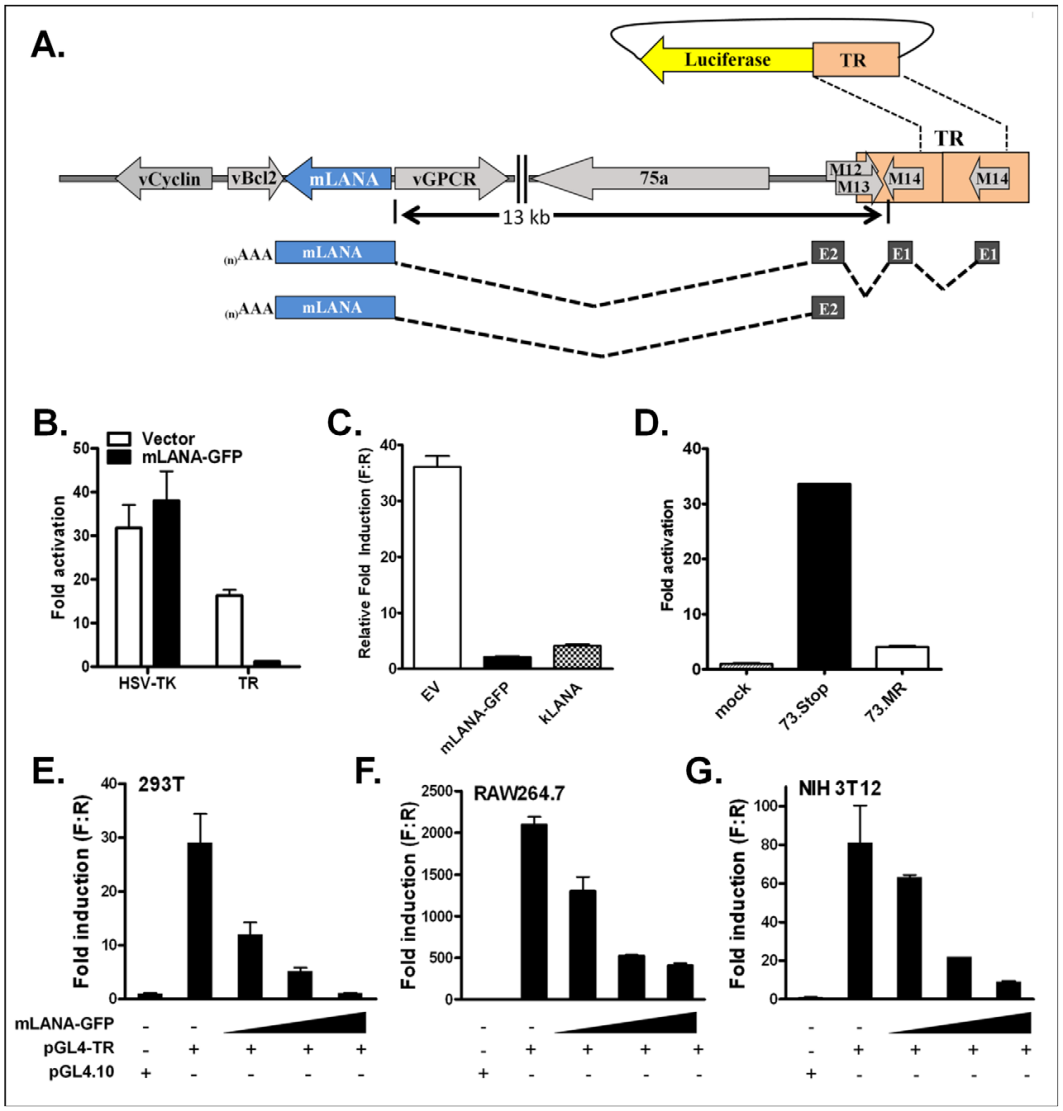
mLANA represses a promoter that initiates transcription within the MHV68 terminal repeat (TR). (A) Schematic illustration of mLANA gene transcription arising from 2 distinct promoters, one in the unique region adjacent the MHV68 TR, and the other within the TR. A firefly luciferase reporter construct was generated in which a single copy of the MHV68 TR (Not I fragment) was cloned upstream of the reporter gene. This reporter plasmid was used in the assays described in panels B to G. (B) A reporter vector containing one copy of the NotI fragment of the TR (pGL-TR) exhibits significant activation over background in HEK 293T cells. Co-transfection of an mLANA expression vector (pMSCV-73GFP) significantly represses the TR-associated promoter activity, but does not repress the HSV-TK promoter. (C) Co-transfecting an equivalent amount of KSHV LANA expression plasmid represses TR transcription as well as mLANA. (D) Infection with MHV68 further activates TR-driven luciferase activity 5-fold, and in infection with an mLANA-null virus (73.Stop), the TR-driven luciferase reporter construct is activated further still (33-fold), indicating a role for mLANA in controlling transcription from the TR during lytic infection. (E–G) The MHV68 TR promoter(s) is/are active in various cell lines and mLANA represses it in a dose-dependent manner. (E) NIH3T12 murine fibroblasts, (F) RAW264.7 murine macrophage, and (G) HEK 293T human epithelial cells were co-transfected with 100 ng pGL-TR or the empty reporter vector (pGL4.10), along with 0, 5, 50, or 500 ng of an pMSCV-73GFP, each balanced to a total of 500 ng with empty expression vector (pMSCV-EFGP). Cell lysates were assayed for luciferase expression at 48 h. The TR promoter(s) is/are active in each cell type, and activity is diminished, in a dose-dependent fashion, with the addition of mLANA expression. Each set is performed in triplicate, and data are presented a ratio of normalized RLU (ratio of firefly to *Renilla* luciferase) over the empty expression vector (pGL4.10) normalized RLU.

In previous work, our lab showed that the mLANA-null virus (73.Stop) displays dysregulated gene expression, characterized by hyper-expression of all temporal classes of viral genes at early time points post-infection of murine fibroblasts compared to the genetically-repaired marker rescue virus (73.MR), as measured by western blot [27] and RT-PCR (Paden and Speck unpublished observation). Included in these hyper-expressed genes is the *gene 73* transcript, which encodes mLANA. It is known that at least one of the promoters for mLANA expression is contained within the terminal repeat, as identified *in vivo* from infected cells [29] (see schematic diagram of mLANA transcripts shown in Fig. 1A). It is also known that the KSHV LANA can bind DNA in the terminal repeat of the KSHV genome [16]–[18], [30], and the KSHV LANA can modulate expression of a variety of genes [24]. Taken together, we reasoned that mLANA is likely directly regulating transcription of viral genes, including the mLANA promoter within the terminal repeat of MHV68. To test this hypothesis, we cloned a complete copy of the MHV68 terminal repeat (using NotI, which cuts only once within the terminal repeat, liberating a 1213 bp restriction endonuclease fragment) into the reporter vector pGL4.10 upstream of the firefly luciferase gene (pGL-TR). The pGL-TR plasmid was co-transfected into cell lines with either the control parental expression vector or a mLANA expression vector, and luciferase activity assessed at 48 hours post-transfection (Fig. 1B).The pGL-TR reporter vector exhibited significant activity in the absence of mLANA expression. Furthermore, luciferase expression from the pGL-TR plasmid was potently inhibited in the presence of mLANA-GFP (pMSCV-73GFP) (Fig. 1B). Notably, the observed repression of activity mediated by mLANA appeared specific since co-transfecting the mLANA-GFP expression plasmid had no effect on reporter activity driven by the HSV-TK promoter (Fig. 1B).

Because there are significant differences in the N-terminus of KSHV LANA and mLANA, we tested whether there is enough conservation of sequence and structure between the KSHV and MHV68 LANA homologs to extrapolate findings on mLANA to the human virus. As such, we assessed whether KSHV LANA can functionally substitute in the MHV68 TR repression assay. We co-transfected HEK 293T cells with the MHV68 TR reporter construct (pGL-TR) and either empty expression vector, the MHV68 LANA expression vector, or the KSHV LANA expression vector (Fig. 1C). Importantly, we observed (using an equivalent amount of input of KSHV LANA or mLANA expression vector) that both the KSHV LANA and mLANA strongly repressed transcription from the MHV68 TR. This indicates that though there is significant amino acid sequence divergence between these homologs, along with the fact that the known KSHV LANA binding sites are not precisely conserved in the MHV68 TR, there is enough functional similarity between the two homologs to reveal a conserved transcriptional repression function. Thus, this provides additional confidence that studies on the MHV68 LANA will likely provide insights into KSHV LANA function—particularly with respect to functions that map to the conserved C-terminal domain.

To address whether LANA repression of transcription from the MHV68 TR is detected in the context of MHV68 infection, we introduced the pGL-TR reporter construct into NIH 3T12 fibroblasts by electroporation, followed by MHV68 infection at a multiplicity of 3 PFU/cell (viral infection was initiated once the cells had adhered to the culture plates approximately 12 hours post-electroporation). Cells were harvested 18 to 24 hours post-infection and assayed for luciferase activity (Fig. 1D). Comparing luciferase induction to cells that were mock infected, we observed that WT virus (73.MR) infected cells exhibited ca. a 5-fold increase in TR-driven luciferase activity, indicating that overall transcription arising from the TR is stimulated by viral infection. However, cells infected with the mLANA-null mutant virus (73.Stop) exhibited significantly higher levels of TR-driven luciferase activity (30- to 35-fold induction) compared to mock infected cells. The latter provides evidence that mLANA plays an important role in regulating transcription initiating from the TR and likely plays a critical role in preventing over-expression of mLANA during infection of permissive fibroblasts (Fig. 1D).

We extended these initial studies to assess mLANA repression of the MHV TR promoter(s) in several different cell lines. Notably, we observed that the promoter contained in the TR had significant activity in a variety of cell types, including NIH 3T12 mouse fibroblasts (Fig. 1E), the murine macrophage cell line RAW264.7 (Fig. 1F), and the human embryonic kidney cell line HEK 293T (Fig. 1G). Furthermore, when the pGL-TR reporter plasmid was co-transfected along with increasing amounts of the mLANA-GFP expression vector, in all cell lines examined the expression of luciferase decreased in a dose-dependent manner (Fig. 1, panels E–G). Taken together these data demonstrate that mLANA represses transcription initiating from the TR—an observation that is consistent with the observed hyper-transcription of mLANA transcripts (as well as other viral genes) in the absence of mLANA expression in cells infected with the mLANA-null mutant virus (73.Stop) [27].

### Identification of DNA sequences required for mLANA repression of MHV68 TR-driven luciferase expression

**Figure 2:**
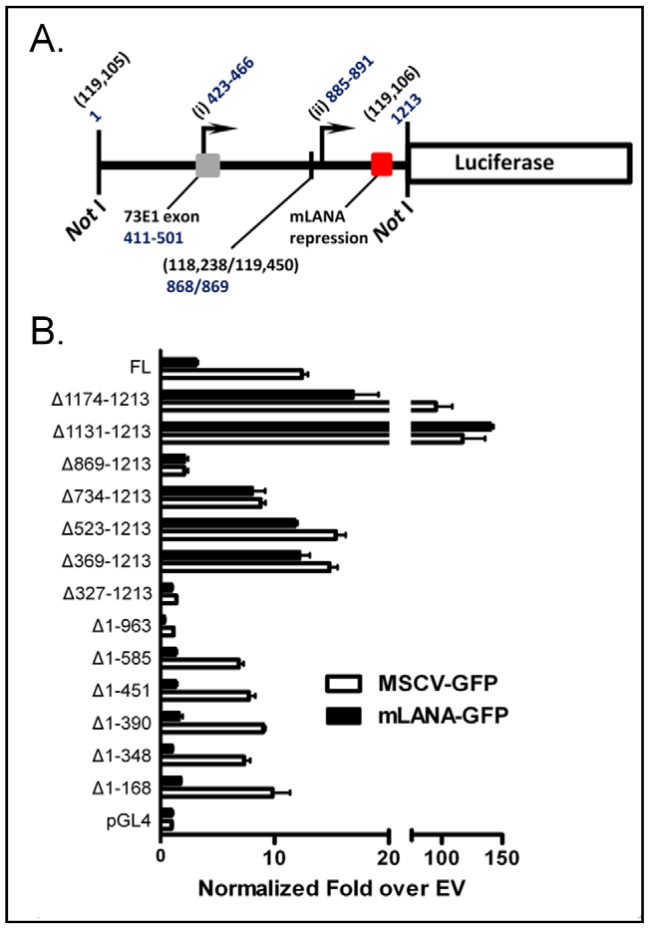
Deletion analysis of TR reveals DNA sequence required for mLANA-mediated repression. (A) Schematic of pGL-TR, noting the numbering scheme with regard to the NotI site. Coordinate 1 is corresponds to MHV68 WUMS [31] bp 119,105, proceeding in order towards the right end of the unique sequence. Coordinate 894 is the end of one TR unit fused to the beginning of the next. Indicated on the diagram are two known sites of transcription initiation: (i) (73p1) starts transcription between coordinates 423–466 (118,683–118,640) depending on cell type [29], [32] and the partial exon splices to the full 73E1 exon (118,695–118,605) in the next repeat unit; (ii) initiates transcription of one species of ORF75a beginning between coordinates 885–891 (118221–118216) [32] (promoter elements are likely shared between ORF75a and ORF73 transcripts that initiate at 73E2 [29], and in distal copies may splice into 73E1). (B) Serial deletions of the TR were made in the pGL-TR vector by PCR and are named accordingly. Each TR deletion, including the full-length TR (FL) was co-transfected into 293T cells, along with either an mLANA-GFP or empty GFP expression vector, as in Fig. 1, in triplicate. Here, data are normalized by taking the ratio of each deletion construct over its respective pGL4.10 empty reporter control, setting pGL4.10 in each case to 1. Deleting the 3′ end gives a large uptick in activity, which is still repressed by mLANA, until the second deletion removes mLANA sensitivity. 5′ deletions had no effect on mLANA sensitivity.

**Figure 3:**
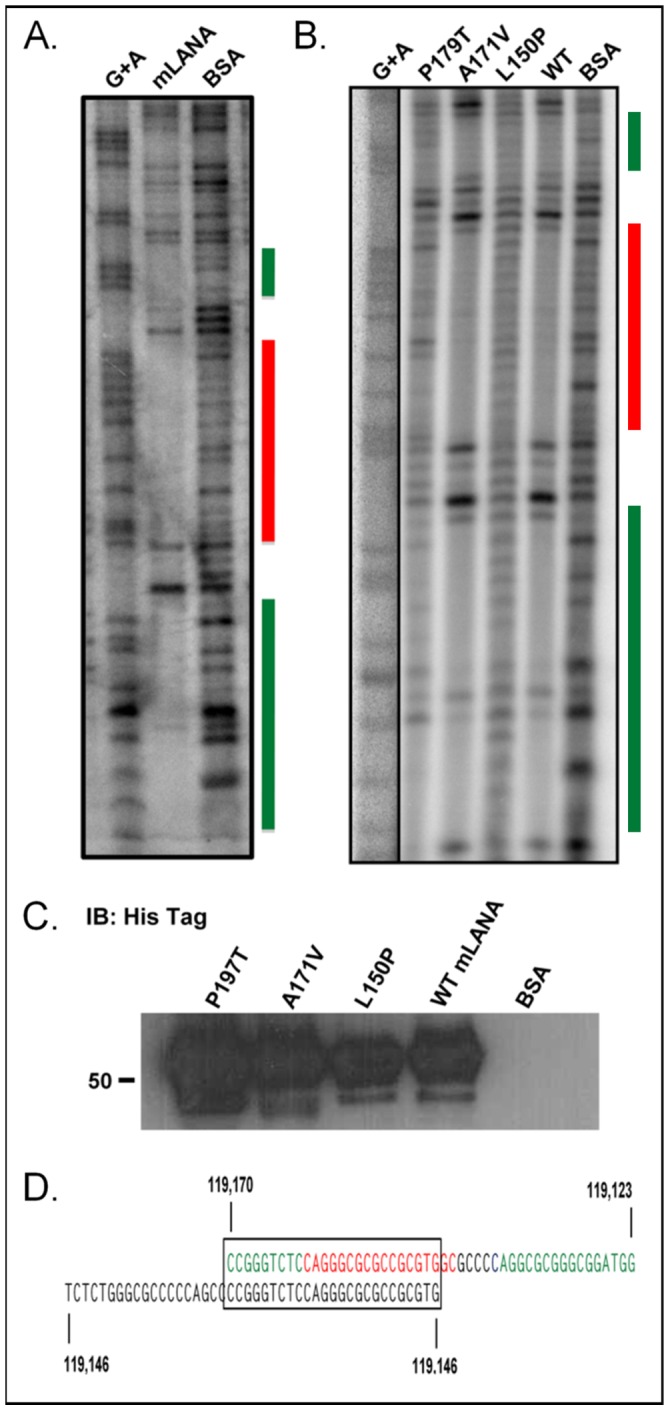
DNaseI footprinting of the terminal repeat with recombinant mLANA shows sequence-specific DNA binding. (A) After a binding reaction with the terminal repeat NotI fragment from pGL-TR, which was radiolabeled at the 3′ end, and either 50 µg purified mLANA-6XHis+50 µg BSA, or 100 µg BSA alone, as a negative control, the protein/DNA mix was subjected to DNaseI digestion, and after DNA recovery, loaded onto an 8% denaturing polyacrylamide gel. The cluster of three protected regions is denoted by the colored bars to the side. This image is representative of several repeats, as well as the result of optimizing mLANA, BSA, and TR concentrations. (B) DNase I footprint analysis of the mLANA mutants P179T, A117V, and L150P, along with WT mLANA and BSA, performed as in (A). The mutants which do not repress transcription do not bind DNA. (C) Immonoblot of recombinant mLANA-6XHis affinity-purified from *E. coli*. Protein was run on an SDS-PAGE gel, transferred to nitrocellulose and probed with an anto-His tag HRP-conjugated antibody. (D) The sequence protected in the footprint analysis is shown here, deciphered using the G+A tracks and color coded, reflecting the colored bars in (A). Below the protected sequence in black is the sequence identified in Fig. 2 as being required for mLANA-sensitivity. The box shows the regions that overlap, and the coordinates are genomic coordinates from GenBank U97553.

To determine what sequences within the MHV68 TR are involved in mediating mLANA repression we took two independent approaches: (i) assaying mLANA repression of a panel of TR deletion constructs (Fig. 2); and (ii) DNase I footprinting of the TR using recombinant mLANA protein (Fig. 3). First, nested deletions from either end of the TR NotI restriction endonuclease fragment were generated and cloned into the pGL4.10 luciferase reporter vector (Fig. 2B). Notably, the TR is cloned into the reporter vector with respect to the luciferase open reading frame in reverse orientation from the annotated sequence [31] (GenBank U97553) (i.e., the orientation of the TR fragment is consistent with promoter activity observed in the context of the viral genome that would lead to transcripts extending into the righthand end of the unique region of the MHV68 genome, such as previously characterized for *gene 73* and *gene 72* transcripts [29], [32])(Fig. 2A). As noted above, the NotI fragment in the reporter vector is a 1213 bp fragment corresponding to bp 119,105 to bp 118,238, and continuing from bp 119,450 to bp 119,106 (the junction between copies of the TR is at bp 118,238/119,450 as defined from the complete sequences of the WUMS MHV68 isolate [31]. To simplify the discussion of mapping TR sequences involved in mediating mLANA repression, we have renumbered the NotI TR fragment as nucleotides 1–1213 as shown in Fig. 2A.

TR deletion constructs were co-transfected into HEK 293T cells with either an mLANA-GFP expression vector or the parental empty expression vector, and assayed for luciferase activity (Fig. 2B). Data were normalized as fold over the pGL4.10 empty reporter vector controls. Deleting sequences from the upstream end of the TR (bp 1–585) had little impact on either basal promoter activity or the capacity of mLANA to repress activity. Notably, deleting the sequences from bp 390 to 451, which had no discernible impact on luciferase activity, removed the region encoding one of the previously characterized LANA promoters [transcription initiation site (i) in Fig. 2A] indicating the presence of other transcriptional start sites within the MHV68 TR. Indeed, deletion of the sequences from bp 585 to 963 resulted in a nearly complete loss of reporter gene activity (Fig. 2B, compare activities of Δ1–585 and Δ1–963 reporter constructs), implicating sequences within this region as critical for the observed activity in the absence of upstream TR sequences [e.g., transcription initiation site (ii) shown in Fig. 2A]. That other sites within the TR can be involved in driving luciferase expression was further underscored by analysis of the nested downstream deletions where the Δ369–1213 deletion exhibited basal activity as high as the full length reporter construct (Fig. 2B). This deletion removes both of the previously mapped transcription initiation sites within the TR [sites (i) and (ii) shown in Fig. 2A]. However, as noted by Coleman et al, there is a distinct AT-rich sequence 5′ of the 73E1 start site. When the AT-rich sequences (bp 328–350, or MHV68 genomic coordinates118,778–118,756) are removed in the Δ327–1213 TR reporter construct, basal activity for this promoter is lost. Thus, it appears that there are several potential transcription initiation sites within the TR whose contribution to the observed luciferase activity are likely context dependent (i.e., the presence or absence of other TR sequences).

Deletion of TR sequences from bp 1174 to 1213 resulted in a significant increase (ca. 8-fold) in basal luciferase activity (Fig. 2B), revealing the presence of a potent negative cis-element in this region of the TR. Notably, the Δ1174–1213 reporter construct was repressed by mLANA to the same extent as the full length TR reporter construct (Fig. 2B). However, a further deletion which removes the sequences from bp 1130–1174 resulted in the complete loss of mLANA repression. The lack of mLANA repression was also observed with all the other downstream nested deletion mutants that lacked the sequences from bp 1130–1174 (Fig. 2B). Thus, within the 1213 bp TR fragment, a single region was mapped that mediates mLANA repression of TR-driven luciferase activity.

To assess whether mLANA, like KSHV LANA, mediates repression through directly binding a target site(s) within the TR, as opposed to being dependent on associations with other DNA-binding proteins, we performed DNase I footprinting on the MHV68 TR NotI fragment using recombinant mLANA-6XHis tagged protein purified from E. coli lysates. Using recombinant mLANA affinity purified from bacteria ensured that any mLANA-dependent DNA binding observed would not reflect binding of any mLANA-associated eukaryotic protein(s), but rather DNA binding activity of mLANA. We radiolabeled labeled each end of the TR separately and performed footprinting assays using each of these labeled fragments and varying amounts of recombinant mLANA-6XHis in the presence of BSA, and with BSA alone as a negative control. We found a single region of the TR, composed of a cluster of ca. three distinct sites (flanked by DNAse I hypersensitive sites), that was protected from DNAse I digestion (Fig. 3A). Notably, the region of mLANA binding overlapped substantially with the region identified as critical for mLANA repression of the pGL-TR luciferase reporter constructs (see Fig. 3D). Thus, these data together provide strong evidence that mLANA-mediated repression of the pGL-TR luciferase reporter construct involves direct interaction of mLANA with the identified sequences within the MHV68 TR.

### Mutagenesis of mLANA reveals residues required for repression of transcription

Having established the TR reporter assay as a robust readout of mLANA-mediated transcriptional modulation, we used this assay to screen for mLANA mutants impaired in repressing transcription. To identify the region or regions of mLANA that are important in regulating transcription, we took an approach that afforded an unbiased analysis of the entire mLANA coding sequence. Low-fidelity/error-prone PCR was used to amplify the mLANA ORF, using the polymerase Mutazyme II (Stratagene) and PCR conditions that favor one to two errors per amplified template. The resulting PCR products were then cloned into an expression vector to generate C-terminal GFP fusion proteins, and single colonies were grown up for DNA isolation. These mutated mLANA-GFP plasmids were subsequently transfected into HEK 293T cells in 96 well plates, along with the pGL-TR reporter vector and the internal control phRL-SV40 which expresses *Renilla* luciferase. Forty-eight hours post-transfection each well was examined for GFP expression, to identify and exclude those wells in which either a nonsense mutation was incorporated into the mLANA coding sequence or that the mutation(s) introduced led to the formation of an unstable fusion protein. Thus, those wells with robust GFP expression were subsequently analyzed for luciferase activity to identify clones that were incapable of inhibiting transcription from the MHV68 TR. As such, those clones that were GFP-positive and luciferase-high were regrown in *E. coli* and retested for loss of transcriptional repression. Those clones in which the phenotype was confirmed were then sent for DNA sequence analysis to determine the mutations introduced into the mLANA coding sequence.

**Figure 4:**
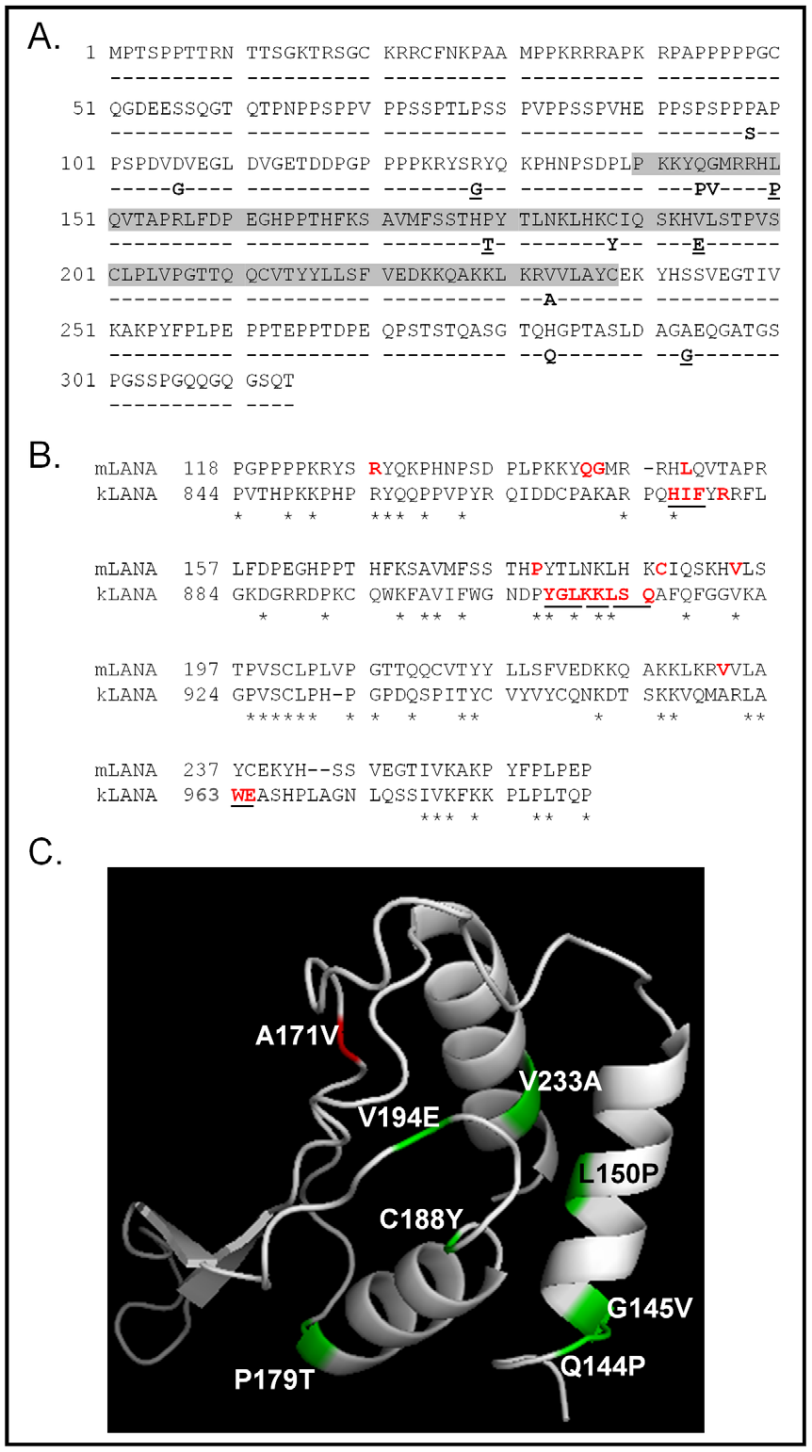
Random mutagenesis of mLANA reveals residues important for transcriptional repression. Loss of function mutants generated at random by error-prone PCR and defined by their failure to repress TR-luciferase reporter activity are shown here. (A) The mLANA primary amino acid sequence is shown, and underneath, each amino acid difference shown represents an amino acid changed in a mutant that had lost its capacity to repress transcription. Highlighted in grey is the region that has homology to other LANA proteins and is the domain that is predicted to fold like the EBV EBNA1 DNA binding domain, as shown in panel C. Underlined are mutations that were introduced into the MHV68-YFP BAC in subsequent experiments. (B) Sequence alignment of the conserved C-terminal domains of mLANA and KSHV LANA (kLANA). Shown in red are mutations in mLANA and kLANA that diminish LANA-mediated repression of transcription (those mutations introduced into kLANA that altered more than 1 residue are underlined). (C) The PHYRE protein structure prediction algorithm modeling of the conserved C-terminal domain (shaded in grey in panel A) of mLANA based on the solved structure of the EBNA-1 DNA-binding domain [33], [34]. This depiction of the predicted three-dimensional structure of the mLANA conserved C-terminal domain has highlighted in green mutations that ablate the transcription repression function of mLANA. Also shown in red is a mutation, A171V, that does not impact mLANA transcriptional repression (has wild type phenotype) and was used in subsequent experiments as a negative control mLANA mutant.

Approximately 1500 clones isolated from the mutagenesis protocol were screened, and 14 clones (∼0.93%) displayed expression and a robust enough change in phenotype from wild-type mLANA to warrant further analysis (Table 1). Though the random mutagenesis strategy was not designed to be an exhaustive search, we found that the majority of the mutations fell within the domain of mLANA (highlighted in grey in Fig. 4A) that is relatively well conserved among the sequenced LANA homologs. Notably, 3 of the clones recovered contained 2 missense mutations in the mLANA coding sequence, while a fourth clone had incorporated 3 missense mutations (Table 1). The remainder of the mLANA mutants analyzed harbored a single missense mutation (Table 1). For the complex mutants, several individual point mutations were introduced into mLANA to pinpoint the residue involved in loss of transcriptional repression.

**Table 1:** Repression activity of mLANA mutants in the MHV68 TR-driven luciferase reporter assay.

Clone	Mutation	Activity*	Dimerize
3C4	S86L/Q144P/S300P	−	nd
13C2	P98S	+	nd
2E4	D106G	−	nd
14A9	D106V	+	nd
3H8	R128G	−	nd
15G3	G145V	−	yes
16A10	L150P	−	yes
3D7	A171V/Q308P	−	yes
1C7	P179T/E260D	−	yes
1G5	C188Y/S273F	−	yes
4A6	V194E	+	yes
2C11	V233A	−	nd
16G4	H283Q	−	nd
16A3	A293G	−	nd
3C7b	Q144P	−	nd
1C7a	P179T	−	yes
1G5a	C188Y	−	nd
4H12	P72H	+++	nd
16F9	V70M	+++	yes
3H7	D111E	+++	nd
3D7a	A171V	+++	yes

* −, 0–20% repression; +, 20–50% repression; ++, 50–70% repression; +++, 80–100% repression.

A number of these mutations were changes in residues conserved between KSHV and MHV68 LANA proteins, and others were in residues that share similar charge or hydrophobicity. Notably, many of these randomly-generated mutations overlap with regions of KSHV LANA targeted by Han *et al.* to disrupt LANA DNA binding (Fig. 4B). Shown in red in Fig. 4B are mutations that attenuate mLANA-mediated transcriptional repression in our hands (MHV68_LANA) and mutations in KSHV LANA that result in ≤50% of WT KSHV transcriptional repression [23].

To assess how the mutations that disrupt mLANA repression are distributed on a three-dimensional model of mLANA (to date there is no available crystal structure of any LANA homolog), we used the Phyre (Protein Homology/analogy Recognition Engine) 0.2 algorithm and online search tool to indentify similar structures. We queried the Phyre tool (http://www.sbg.bio.ic.ac.uk/~phyre/) using the full-length amino acid sequence of mLANA and searching against profiles of proteins with known structures [33]. This algorithm determined that the top hit was for the shaded region of the mLANA sequence shown in Fig. 4A, which matched most closely to the DNA-binding domain (DBD) of the Epstein-Barr virus nuclear antigen-1 (EBNA-1) protein—the presumed functional homolog (encoded by the lymphocryptovovirus subclass of gammaherpesviruses) of the rhadinovirus LANA proteins [34], [35]. Notably, this region of EBNA1 shares almost no homology and little similarity to the primary sequence of mLANA. This is similar to what was found using KSHV LANA as a query sequence in an older 3D-PSSM algorithm [36]. Using this model, we mapped the loss of repression mutations in this region on the EBNA-1 DBD/mLANA hybrid model (Fig. 4C). The sequence shaded in grey in Fig. 4A is shown in the model, with the mutations colored in green. For reference, an additional mutant we generated that does not disrupt activity (A171V) is colored in red (Fig. 4C). From this analysis we hypothesized that the mLANA loss of function mutations identified in this region disrupt DNA binding, either by causing the β-barrel structure of the core DNA-binding domain [37] to misfold slightly, misaligning contact residues, or by directly mutating a contact residue.

### Mutations in the predicted mLANA DNA-binding domain disrupt mLANA-DNA interaction

**Figure 5:**
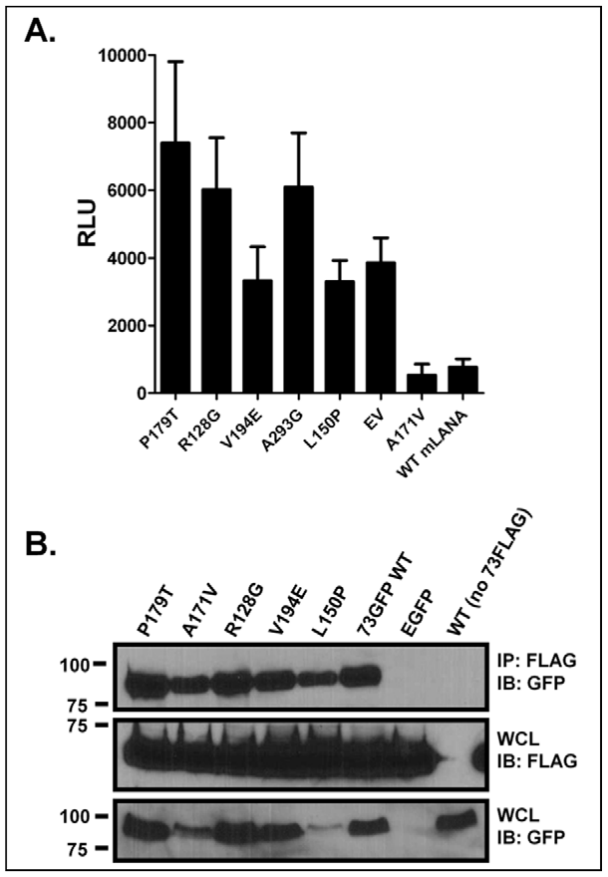
mLANA mutants lose transcription repression function, but not the capacity to dimerize. (A) Analysis of mLANA mutants that exhibited diminished repression of the MHV68 TR-driven luciferase activity. The mutants chosen, with the exception of the A171V mutant, have diminished capacity to repress transcription from the TR promoter(s). Data are expressed as relative light units. Comparisons to WT mLANA, with respect to the effectiveness of repressing transcription, were assessed using a student's t test: *, P<0.05; **, P<0.01; ns, not significant. Differences between each non-repressive mutant and empty vector (EV) were not significant (P>0.05). (B) Expression vectors for WT mLANA-3XFLAG, along with WT mLANA-GFP or the indicated mLANA-GFP mutants were cotransfected into 293T cells. Lysates were collected and co-immunoprecipitated using FLAG-agarose beads and the recovered complexes resolved by SDS-PAGE. Control pulldowns included EGFP with mLANA-3XFLAG and WT mLANA-GFP and the empty FLAG tag expression-vector, which fail to co-immunoprecipitate. Shown are anti-GFP blots for the pulldown and whole cell lysates, as well as an anti-FLAG blot for the whole cell lysates.

Based on the EBNA-1 structure prediction, we chose six mutants to further characterize. The mutants chosen were: (i) P179T, which is predicted to be in helix 2, considered part of the core DNA-binding region; (ii) A171V, which would be in the turn between the flanking and core domains and has no measured effect; (iii) R128G, which would be in or near the minor groove contact loop of the flanking domain; (iv) V194E, which would be in the turn between the two helices of the core domain; (v) A293G, which is predicted to be downstream of the core DNA-binding domain; and (vi) L150P, which would be part of the flanking domain, near the major groove contact residues. Their effects on repression of the TR-driven luciferase activity are all significantly different than wild-type mLANA, save for the A171V mutant (Fig. 5A). Notably, the relatively small differences in the activities of the mLANA mutants that have lost repression compared to the empty vector (EV) control were not statistically significant (Fig. 5A).

To assess whether or not the mutations we identified that exhibit diminished transcriptional repression activity actually lose the capacity to bind DNA, we generated recombinant protein for three of the mutants, P179T, A171V, and L150P (Fig. 3C). We performed DNase I footprinting on these mutant mLANA proteins, along with WT mLANA, and observed that while there may be subtle differences between the P179T and L150P mutants—both of which fail to repress transcription—neither produced robust protection from DNase I digestion demonstrating decreased DNA-binding (Fig. 3B). In contrast, both the wild-type mLANA and the A171V mutant, which exhibits wild-type phenotype in the pGL-TR luciferase repression assay (Fig. 5A), show robust sequence-specific DNA binding (Fig. 3B). It should be noted that the absence of DNA binding observed with the P197T and L150P mutants is unlikely to reflect inadequate levels of recombinant protein used in the DNAse I footprinting assay, based on SDS PAGE analyses of the purified mLANA proteins (Fig. 3C).

Since it has been shown that the DNA-binding domain and the dimerization domain in EBNA-1 are near each other [34], [37], [38], we wanted to know: (i) if mLANA also forms dimers, and (ii) whether any of the identified mLANA mutants are defective in dimerization, which would likely render them non-functional as it has been shown that KSHV LANA binds DNA as a dimer [30]. To assess dimerization, we generated a construct that expresses mLANA with a C-terminal 3XFLAG epitope tag (p73-3XFLAG) and co-expressed that construct along with five mLANA mutants (expressed as GFP-fusion proteins), WT mLANA-GFP, or GFP alone. After immunoprecipitating with an anti-FLAG antibody, we resolved the complexes by SDS-PAGE and probed immunoblots with an anti-GFP antibody (Fig. 5B). Notably, as expected we observed that WT mLANA-3XFLAG was capable of interacting with and pulling down WT mLANA-GFP, demonstrating formation of mLANA dimers (Fig. 5B). In addition, WT mLANA-3XFLAG was also capable of pulling down each of the mLANA mutants, but not GFP alone (Fig. 5B). Additionally, as a control we co-expressed the empty 3XFLAG vector and WT mLANA-GFP and demonstrated that the anti-FLAG antibody did not immunoprecipitate mLANA-GFP (Fig. 5B). These analyses demonstrate: (i) dimerization of mLANA; and (ii) all of the identified mLANA mutants are able to dimerize with wild-type mLANA. Notably, while the L150P mutant (and to a lesser extent the A171V mutant) were expressed at lower levels, similar levels of mutant protein appeared to be recovered by co-immunoprecipitation of wt mLANA. It is likely that this reflects that the co-immunoprecipitation assay is not quantitative and that antibody is the limiting, leading to similar levels of mLANA dimers being recovered following immunoprecipitation.

### MHV68 LANA DNA-binding and/or transcriptional repression null mutant viruses exhibit a mLANA-null phenotype in vivo

The advantage of using MHV68 to study LANA biology is that: (i) it is straightforward to create mutant viruses, as MHV68 replicates robustly in tissue culture and the viral genome has been cloned into a bacterial artificial chromosome (BAC); and (ii) the virus readily infects naïve laboratory mice, allowing detailed viral pathogenesis studies. For these reasons, we chose six mLANA mutations to introduce into the viral genome to assess the impact that the transcriptional repression and/or DNA-binding functions of mLANA have on the overall pathogenesis of MHV68. To construct the mutant BACs, we employed λRed-mediated homologous recombination using *galK* to facilitate selection in the *E. coli* strain SW102 [39]. The MHV68-YFP BAC [40] was transformed into the *E. coli* strain SW102. Next, the entirety of ORF73 was replaced with the *galK* cassette, flanked by 50 bp arms homologous to the region upstream and downstream of ORF73, and selected on galactose-containing minimal media to create pMHV68.73galK. Clones were verified by restriction digest. PCR products of ORF73 containing each mutation, as well as the 73.Stop mutation [28], and WT ORF73 were introduced into SW102 harboring MHV68.73galK BAC for recombination onto the galK BAC. Resulting colonies were verified by colony PCR and restriction digest. Since there are no diagnostic restriction sites in these point mutants, the ORF73 region of each BAC was further PCR amplified and sequenced (data not shown). The resulting mLANA-transcription-repression-null mutant viruses (73TRN) are designated 73.P179T, 73.R128G, 73.V194E, 73.A293G, and 73.L150P, along with 73.A171V, 73.Stop-2, which contains a stop codon near the beginning of the ORF identical to 73.Stop [28], and the genetically-repaired 73.galKMR.

**Figure 6:**
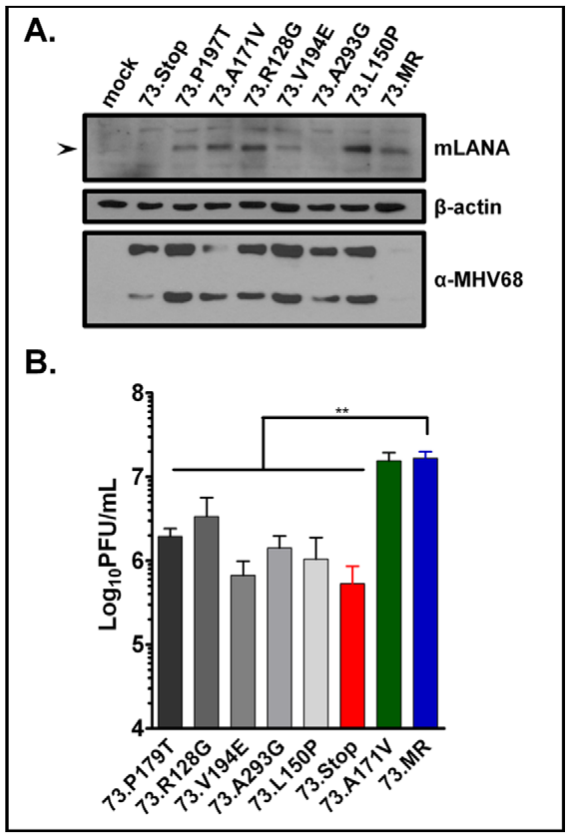
mLANA mutants that have lost the capacity to repress transcription from the MHV68 TR-driven luciferase reporter construct (MHV68-73TRN mutants) display similar growth kinetics and virus output as the mLANA null mutant virus (73.Stop virus). Six mLANA mutants were introduced into the MHV68 genome using BAC recombineering. (A) After infection of NIH3T3 fibroblasts with each MHV68-73TRN virus at an MOI of 3 PFU/mL, cell lysates were harvested at one and seven hours post-infection. The lysates were immunoblotted with rabbit mLANA antiserum, mouse MHV68 antiserum, and anti-β-actin antibodies. (B) NIH3T3 fibroblasts were infected at an MOI of 0.001 PFU/cell, and cells and supernatants were harvested at day 7 post-infection and titered on NIH3T12 cells. The resulting data shows that the MHV68-73TRN viruses grow to titers similar to that of 73.Stop, about 10-fold lower than the genetically-repaired 73galkMR virus. **, P<0.01.

To ensure that our mLANA mutants would express in the context of infection, we infected NIH3T3 cells at an MOI of 3 PFU/cell and collected lysates at seven hours post-infection. We readily observed mLANA expression by immunoblot, using a rabbit polyclonal anti-mLANA antibody, in all mutants except for A293G (the basis for failure to detect the latter mutant is unclear) (Fig. 6A). Further, a phenotype of 73.Stop virus we previously observed was dysregulation of lytic gene expression, which is manifested as hyperexpression of all lytic genes. Each mutant also displayed expression of antigens detectable by lytic antiserum, except for the 73.galK marker rescue (73.MR) and 73.A171V viruses, which behave as WT (Fig. 6A).

We have previously shown that mLANA is required for efficient replication in tissue culture following low MOI infection—the mLANA-null virus 73.Stop showed a 1- to 2-log defect in output virus from permissive fibroblasts upon low MOI infection (between 0.05 and 0.001 PFU/cell) compared to the genetically repaired 73.MR virus [27]. To determine whether mLANA DNA binding is required for efficient virus replication, we infected NIH3T3 fibroblasts with each 73TRN virus at an MOI of 0.001 PFU/cell. Cells and supernatants were collected by freeze/thaw lysis seven days post-infection and titered on NIH 3T12 fibroblasts. We determined that the 73TRN viruses, like 73.Stop, exhibited a significant decrease in output titers over the course of several rounds of virus infection, whereas the 73.A171V mutant was equivalent to 73.galK marker rescue (73.MR) virus (Fig. 6B).

### In vivo analyses of 73TRN viruses demonstrate a requirement for mLANA DNA-binding domain

**Figure 7:**
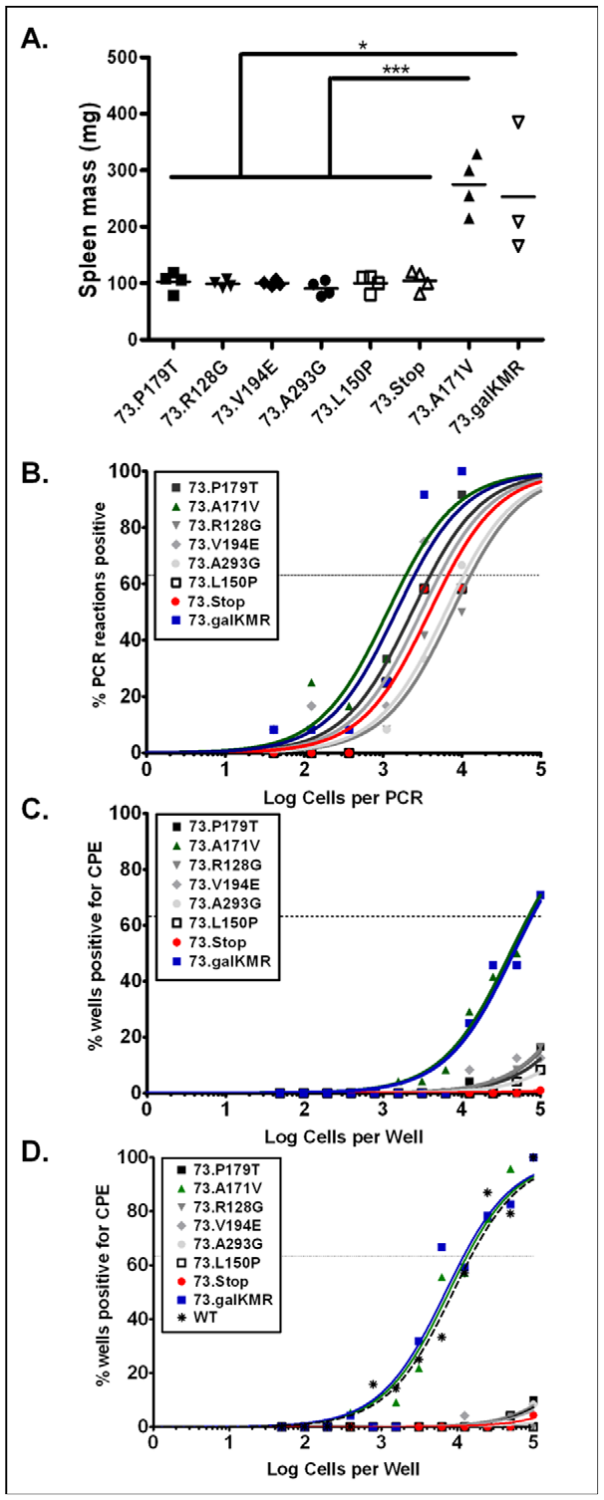
Intraperitoneal infection of C57Bl/6 mice with MHV68-73TRN mutants reveals mLANA DNA-binding/transcriptional repression function is essential for reactivation from splenocytes. Groups of 3–4 mice were infected intraperitoneally with 1000 PFU of each MHV68-73TRN virus. Eighteen days post-infection mice were sacrificed, and (A) spleens were weighed, (B) the frequency of infected splenocytes was determined by limiting-dilution PCR, and (C) the frequency of cells that reactivate virus was determined by the limiting-dilution CPE assay. In all cases the MHV68-73TRN viruses behaved similar to the mLANA null mutant 73.Stop. (D) Similarly, mice infected intranasally with 1000 PFU each virus were assayed for cells reactivating virus 18 days post infection. *, P<0.05, ***, P<0.001.

As with the in vitro replication defect known for the mLANA-null 73.Stop virus, we have previously shown that MHV68 requires mLANA expression for efficient reactivation from latently-infected cells [21]. To examine whether the transcription repressor function of mLANA is required for reactivation from latency, we used the 73TRN mutant viruses to infect mice and assay for establishment of and reactivation from latency. Each 73TRN virus was used to infect groups of C57Bl/6 mice intraperitoneally with 1000 PFU of virus per mouse. The intraperitoneal route was used since the 73.Stop virus is capable of establishing latency in immunocompetent mice via this route and not by the intranasal route [21], [28], [41]. Eighteen days post-infection, mice were sacrificed and spleens and peritoneal exudate cells were harvested and subjected to limiting dilution PCR (Fig. 7B) and an ex vivo limiting dilution reactivation assay (Fig. 7C). Somewhat surprisingly, we found that every mLANA mutant that was null in the TR transcription repression assay when introduced into MHV68, displayed limited splenomegaly, similar to the mLANA-null mutant (73.Stop) (Fig. 7A), and a reactivation phenotype comparable to the mLANA-null mutant (Fig. 7C) (i.e., these virus-infected cells displayed no detectable reactivation in the limiting dilution assay). Under these experimental conditions, the 73.Stop mutant also displays a slight decrease in the frequency of latently infected cells—ca. 3 to 5-fold lower than the MR virus—which is borne out here in each of the 73TRN mutant viruses (Fig. 7B). These data point to the conclusion that mLANA DNA-binding and/or transcriptional repressor functions play a critical role in viral pathogenesis.

Intranasal infection of mice with these mutant viruses also led to an ablation of reactivating virus from the spleen (Fig. 7D), not surprising based on intraperitoneal infection results, as well as previous data regarding 73.Stop intranasal infection [28], [41].

## Discussion

Here we report that mLANA can function to repress transcription driven from the MHV68 TR via binding to a specific site within the TR. In addition, we report the development of a rapid method for generating and screening loss-of-function mLANA mutants, and have used this approach to identify and characterize mLANA mutants that have lost the capacity to repress transcription from the MHV68 TR. Finally, we have used this information to generate mLANA mutants in the context of the viral genome and shown that the role of mLANA in: (i) virus replication in permissive fibroblasts in vitro; (ii) regulation of viral gene expression during entry into the lytic cycle; and (iii) virus reactivation from latency, are all dependent on mLANA DNA binding. Indeed, all the mLANA mutants characterized which do not repress TR transcription exhibit a phenotype indistinguishable from the mLANA-null mutant virus. Thus, these studies on mLANA repression of transcription highlight the role of the mLANA DNA-binding domain and its function during viral infection.

The promoters and regulatory regions within the terminal repeat identified here are consistent with various elements previously mapped by others [29], [32]. There exist at least two promoters within the terminal repeats, 73p1 [29], which initiates transcription of exon 1 of gene 73, and an ORF75a promoter (which shares cis-elements with the 73p2 promoter and may also drive transcription of ORF73 in vivo [32], [42]), identified during reactivation from infected splenocytes, as well as during de novo infection of fibroblasts. Results from our deletion analysis are consistent with data from these studies identifying a promoter driving a transcript initiating between bp 118683–118640. Interestingly, deleting these coordinates did not ablate transcription from this area, but when we deleted the A/T-rich region between bp 118780–118738 (TR coordinates 327–369), luciferase activity was reduced to background levels, indicating that this region (−73 relative to the most 5′ TSS) is critical for transcribing exon 1 of gene 73. The deletion analysis also confirms the existence of a second promoter which initiates a transcript between bp 119434–119429. Note that the latter sequence is homologous to bp 118221–118216 in the viral genome, which is within the short 27 bp partial TR sequence at the righthand end of the MHV68 unique sequence immediately adjacent to the first complete copy of the TR, as defined by the sequence of the MHV68 WUMS sequence [31] [Fig. 2; shown as transcription initiation site (ii)].

Importantly, all of the regions that appear to be involved in TR-driven reporter gene activity lie upstream of the mLANA-binding site(s), which were mapped at genomic coordinates 119169–119122. In this context, mLANA potently repressed reporter gene activity. KSHV LANA has been shown to bind to sequences in the KSHV TR and to have a similar negative effect on transcription from promoters placed near the TR in a reporter vector [16]. Unlike the MHV68 TR, where one copy of the TR is sufficient to observe both promoter activity and its efficient repression by mLANA, the KSHV TR has not been shown to contain promoter activity on its own, and multiple copies of the TR are required to observe LANA repression of a heterologous promoter [16].

Consistent with our observation that mLANA can repress transcription arising from the TR, it is important to note that the presence of mLANA causes a downregulation of ORF73 transcript levels during virus infection in permissive fibroblasts, as detected by RT-PCR of the ORF73 coding exon. Similarly, in a study of rhesus rhadinovirus (RRV) LANA, many observed viral transcript levels, including ORF73, were elevated during infection with a LANA null RRV mutant [43]. These studies are seemingly contrary to what has been observed with respect to the impact of KSHV LANA in transient transfection analyses, in which LANA has been shown to upregulate its promoter [16], [24], [44]. However, in line with observations with MHV68 and RRV, a KSHV LANA null mutant virus does display hyperexpression of viral replication-associated genes, although the authors did not assess levels of LANA transcripts [45]. Thus, the differences between the behavior of the LANA encoded by RRV and MHV68 and KSHV LANA may simply reflect a paucity of data regarding transcription of LANA during infection. Notably, with respect to this issue, there have not been any studies published demonstrating KSHV LANA transcripts initiating from the terminal repeats. Similarly, there is very little data on the MHV68 LANA promoter located in the unique region of the viral genome [32], [46]. As such, this underscores a need for further careful analyses of the role of LANA homologs in regulating transcription of ORF73, both during the establishment of latency and acute virus replication.

DNA-binding domains have been identified in KSHV LANA [16], [23], [25], [30] and EBV EBNA-1 [38], [47], [48]—the latter appearing to be the functional homolog of the rhadinovirus LANA proteins. We have shown here that mLANA, like KSHV LANA and EBNA-1, physically interacts with DNA in a sequence-specific manner. It is of note that we identified this sequence using two distinct assays, with both approaches identifying the same region of the MHV68 TR as being required for both mLANA DNA binding and repression of TR-driven transcription. Briefly, the TR deletion assay identified the DNA sequence between bp 1130 and 1174 of the TR NotI fragment as required for mLANA-dependent repression. DNase I footprinting analyses of both ends of this TR fragment identified a 48 bp protected region that overlaps with the 44 bp region identified from the analyses of TR-Luciferase deletions constructs. KSHV LANA footprinting of KSHV TR sequences shows a similarly-sized protected region [30]. Further analysis showed that the large footprint was actually two distinct binding sequences, being occupied simultaneously by a LANA multimer [30]. We suspect mLANA binds DNA in a similar fashion, however, further work will be needed to confirm this experimentally.

Notably, with respect to the mLANA DNA binding sites, there is some homology between the MHV68 mLANA TR binding sequence and one of the known KSHV LANA binding sites (LBS) [17], [30]. KSHV LBS1 and the TR binding site share 14 of 16 nucleotides, although there is no significant homology between the MHV68 TR mLANA binding site and KSHV LBS2. However, despite the fact that the exact KSHV LBS1 or LBS2 sites are not present in the MHV68 TR, we observed that KSHV LANA was fully capable of repressing transcription initiated from the MHV68 TR.

The C-terminal portion of mLANA, which is the region with most sequence conservation between rhadinovirus LANA proteins, is predicted to have a tertiary structure similar to that of the EBV EBNA-1 DNA-binding domain. The homologous region of the KSHV LANA is also predicted to share this same similarity with the EBNA-1 DNA binding domain [30], [36]. This argues that studies on mLANA structure and function will likely be informative with respect to KSHV LANA function. Importantly, the majority of loss of repression mutants that were identified from the unbiased mutagenesis screen mapped to this region of mLANA. There has been discussion with respect to the EBNA-1 DNA-binding domain the contribution that the second and third helices (considered the “core” domain), and the first helix (considered a “flanking” domain) in sequence-specific DNA binding [34], [37], [49]. Here, we have identified from a random pool of mutants, loss-of-function mutations that fall within each of these three helices, indicating that all three helices are critical for the formation of a functional DNA-binding domain. Furthermore, our analysis of DNA binding using the mLANA mutants P179T (mutation in helix 2—thought to be in the core region) and L150P (mutation in helix 1—thought to be the flanking region) demonstrated that both mutant proteins failed to protect the mLANA binding site from cleavage by DNase I, further supporting the data obtained from TR-driven luciferase repression assay.

Knowing that the mLANA-null virus exhibits several phenotypes, including: (i) in vitro and in vivo replication defects; (ii) dysregulated viral gene expression; and (iii) failure to reactivate from latently-infected splenocytes [21], [27], [28], we tested whether these phenotypes were dependent on the transcription modulating/DNA binding function of mLANA. Importantly, all of the point-mutant virus analyses revealed that this domain of mLANA is critical for each of these phenotypes. One complicating factor may be that disrupting the DNA-binding capacity of mLANA could have an impact on other functions proposed for LANA homologues aside from transcriptional regulation. For example, mLANA may be involved in viral DNA replication, as it, like EBNA-1, is proposed to be an origin binding protein with the capacity to recruit cellular DNA replication machinery [22], [50]. However, it is unclear from studies of KSHV LANA whether this is required for virus replication and whether it functions in lytic and/or latent replication [51], [52]. Alternatively, it may be that mLANA, which binds very strongly in the terminal repeat region and dimerizes, may facilitate circularization of the genome prior to rolling circle replication or episome formation. Indeed, we have previously reported that in vivo the 73.Stop virus established a life-long infection in mice, but that the viral genome fails to form episomes [21].

mLANA may be utilizing a variety of mechanisms to downregulate transcription, either directly or indirectly. It may directly interfere with transcription, either during the initiation phase or physically during the elongation of a nascent transcript. Another possibility is that mLANA interacts with and recruits proteins that shut off transcription through chromatin modification, similar to KSHV LANA which has been shown to interact with and recruit de novo methyltransferases [53] and members of the mSin3 co-repressor complex [44]. Give the high G+C content of the TR, the cellular transcriptional activator Sp1 may be required for efficient activation of transcription initiation from the TR. It has been shown in EBV that EBNA-1 displaces Sp1 from the TR [54], and it could be that mLANA performs a similar function—mLANA may displace Sp1, or some other cellular transcription-enhancing protein, from key sites, thereby downregulating transcription.

It is proposed that multiple TR copies may function as added attachment sites for strongly tethering the virus genome to the host chromosome via an interaction with host histone protein [36], [55]–[57]. In addition, we propose that multiple copies of the TR (and mLANA binding sites) allow mLANA to control transcription of the latency locus (genes 72, 73, and possibly others) during lytic and latent replication—perhaps before this region becomes methylated during latency (K.S. Gray and S.H. Speck, unpublished observation), which also shuts down transcription. This would be reminiscent to the regulation of EBV EBNA transcripts from the C and W promoters during the early phase of latency—viral promoters that are subsequently silenced by DNA methylation as EBV latency transitions to germinal center and then memory B cells [58]–[60]. It is likely very important to have tight, semi-redundant control over transcription of the latency locus—robust unchecked expression of this locus from KSHV leads to tumor formation in mice [61], [62] and is apparently required for growth transformation/immortalization of B cells by MHV68 [6]. Ultimately, we do not yet have a sufficiently detailed understanding of transcription in this region of the viral genome to appropriately model how mLANA regulation of latency and lytic cycle-associated gene expression impact virus infection.

Finally, the random mutagenesis method we employed in this study may prove useful for developing treatments of gammaherpesvirus-associated diseases. Effective dominant negative forms of EBNA-1 have been identified [63], and small modifications to the mLANA mutagenesis protocol could result in selectively targeting mutations to regions of mLANA other than the DNA binding domain. The latter may lead to the generation mLANA mutants that can occupy LANA binding sites without recruiting additional factors involved in modulating viral gene expression and subsequent cellular transformation. Small molecule inhibitors of EBNA-1 have recently been identified [64], [65] and proposed as therapeutic for treatment of EBV-induced disease. Similarly, the screening approach described here has the capacity to rapidly determine the impact of a large number of individual compounds on mLANA function. We also have the advantage in studying MHV68 mLANA that we can test these strategies in vivo to directly assess effects on pathogenesis and disease. Given the apparent conservation of functions between mLANA and KSHV LANA, such studies could ultimately prove beneficial in the treatment of KSHV-associated diseases.

## Materials and Methods

### Cell lines, transfections and reporter assays

NIH 3T12, RAW24.7, HEK 293T, and MEF (mouse embryonic fibroblast) cells were maintained in Dulbecco's modified Eagle's medium (DMEM) supplemented with 10% fetal calf serum (FCS), 100 U of penicillin per ml, 100 mg of streptomycin per ml, and 2 mM L-glutamine (complete DMEM). Cells were maintained in a 5% CO2 tissue culture incubator at 37°C. MEFs were obtained from C57BL/6 mouse embryos as described previously {Weck, 1996 #372}. Vero-Cre cells were a gift from David Leib. Cells were passaged in DMEM supplemented with 10% FCS and 300 µg of hygromycin B/ml.

Transfections were performed, except where noted otherwise, using the lipid-based reagent TransIT LT-1 (Mirus) according to manufacturer's instructions. For those analyses carried out in 12-well plates (format used for most transfections), 100 ng of reporter and 50 to 500 ng of the relevant expression vector were transfected per well. For some experiments 10 ng of a SV40-driven *Renilla* luciferase, phRL-SV40, was co-transfected with the firefly luciferase reporter vector and served as an internal control. All transfections were harvested 48 h post-transfection. For those transfections carried out in 96-well plates, 50 ng expression vector, 15 ng pGL-TR reporter vector, and 0.2 ng phRL-SV40 were introduced to each well.

For co-immunoprecipitation experiments, 7.5 µg of each expression construct was used. The cells were harvested 48 h post transfection

### Reporter assays

All dual reporter assays were performed using the Dual Luciferase kit (Promega), according to manufacturer's instructions. For single luciferase assays, cells were lysed in an appropriate volume of lysis buffer (25 mM Tris-phosphate, pH 7.8, 2 mM DTT, 2 mM DCTA, 10% glycerol, 1% Triton X-100), and then 10 µL lysate was mixed with 50 µL luciferase assay reagent (1.5 mM HEPES, pH 8, 80 µM MgSO4, 0.4 mM DTT, 2 µM EDTA, 10.6 µM ATP, 5.4 µM Coenzyme A, and 9.4 µM beetle Luciferin) and light units were read on either TD-20/20 luminometer (Turner BioSystems) or a plate reader (BioTek), for large format assays.

### Cloning and plasmids

The mLANA-GFP fusion protein expression plasmid pMSCV-73GFP was described previously [27]. The control plasmid, expressing only EGFP pMSCV-EGFP was made by amplifying the EGFP sequence from pIRES-EGFP using the oligos EGFP_5′_BglII (5′-ggaagatctATGGTGAGCAAGGGCGAGG-3′) and EGFP_3′_EcoRI (5′-tagaattcTTACTTGTAC AGCTCGTCC-3′) and cloning it into the BamHI/EcoRI sites of pMSCVpuro. The mLANA-3XFLAG epitope tagged plasmid was made by PCR amplifying ORF73 sequence from pL3700 [66] using 73-1_BAM (5′- GATCGGATCCCTTGACCCACACCCTTCCTGTGC-3′) and 73-3_nostop_BAM (5′- gatcggatccTGTCTGAGACCC-3′). The PCR product was cloned into the BamHI site in p3XFLAG-CMV-14 (Sigma). The KSHV LANA expression construct was created by excising the LANA sequence from pDD105 [67] and inserting it into the NotI site of pcDNA3.1(+) (Invitrogen).

The reporter plasmid pGL-TR(NotI) (pGL-TR) was created by digesting MHV68 BAC with NotI to obtain the 1240 bp TR fragment. This fragment was cloned into the NotI site of pcDNA3.1(+) (Invitrogen) to create pcDNA-TRnot. The resulting vector was then digested to determine orientation, and then digested with XhoI and EcoRV to liberate a fragment that was then cloned directionally into pGL4.10 (Promega) using the corresponding restriction sites. The TR is oriented towards the luciferase open reading frame in reverse with respect to the annotated sequence.

Serial deletions of the TR were made by amplifying portions of the TR using PCR and then cloning those fragments into the KpnI/BglII sites. The primers used were, for 5′ deletions: TRn-3′BglII (5′-atatagatctGCGCCTGGGGCGCCATGC-3′) in combination with 173F-Kpn (5′-ttaggtacc cccggggcccccacaagcctc-3′), 311F-Kpn (5′-ttaggtacc ccgggggccccgctacgagc-3′), 353F-Kpn (5′-ttaggtacc aagccccgggcccgcccc-3′), 395F-Kpn (5′-ttaggtacc cgtgcccctcccccctgcag-3′), 456F-Kpn (5′-ttaggtacc cctccgggacccgcccacag-3′), 590F-Kpn (5′-ttaggtaccgagggccagagtctgaa ctg-3′), 702F-Kpn (5′-ttaggtacc gagggccagagtctgaactg-3′), or 995F-Kpn (5′-ttaggtaccgtggccgc gctggcctagc-3′); and for 3′ deletions, TRn-5′KpnI (5′-ttaggtaccGGCCGCTAC CGCCCGGGCC-3′) in combination with 311R-Bgl (5′-atatagatctGCTCGTAGCGGGGCCCCCGG-3′), 353R-Bgl (5′-atatagatctCGGGGGCGGGCCCGGGGCTTAC-3′), 526R-Bgl (5′-atatagatctCCCCCTCGGC GGCTCCCCTAC-3′), 737R-Bgl (5′-atatagatctAGGGGGCTGGAGGGGGGAAG-3′), 875R-Bgl (5′-atatagatctCCCCCACCCCCCAAGAAGAG-3′), 1014R-Bg (5′-atatagatctAGCTAGGCCAG CGCGGCCAC-3′), 1161R-Bgl (5′-atatagatctCCCTACCGGGCTGCCGCTAC-3′), or 1205R-Bgl (5′-atatagatctCACGCGGCGCGCCCTGGAG-3′).

The bacterial expression vector pET22b-73dN was generated by amplifying ORF73 from pL3700 using the following primers: 73_5′_ATG2_Bam_2 (5′-gatcg gatccgATGCCTCCTAAAA GACGCC-3′) and 73_3′_nostop_Xho (5′-gatcctcgagTGTCTGAGACCCTTGTCCCTG-3′). The PCR product was cloned into the BamHI/XhoI sites of pET22b (Novagen).

All PCR amplifications for cloning were performed using the high-fidelity Phusion polymerase (New England Biolabs).

### Protein expression and purification

The vectors pET22b-73dN (and also ORF73 mutants) and pRARE (Stratagene) were both transformed into the BL21(DE3) strain of E. coli (New England Biolabs). After bringing 1 L cultures to log-phase growth, expression was induced by the addition of 1 mM IPTG for 5 hours at 37°C. Bacteria were harvested by centrifugation, and lysates were prepared for Ni-NTA purification under native conditions according to the manufacturer's instructions (QIAGEN). Briefly, the pellet was resuspended in lysis buffer, and cells were lysed in the presence of 1 mg/mL lysozyme and by sonication. Following sonication, lysates were treated with RNase A (10 µg/mL) and DNase I (5 µg/mL) prior to clearing. Lysate was incubated with 10 mL Ni-NTA agarose slurry in batch for 1 hour and subjected to column-based washing and elution. 500 µL fractions were collected and analyzed by SDS-PAGE. Those fractions containing the majority of mLANA protein, determined by Coomassie Blue staining, were pooled and dialyzed overnight into buffer BFD (20 mM HEPES, 20% glycerol, 0.1M KCl, 0.2 mM EDTA, 0.5 mM DTT, and 0.5 mM PMSF) and stored at −80°C.

### Radiolabeling and DNase I footprinting

DNase I footprinting was performed essentially as previously described, with minor modifications [68], [69]. Probes were made by digesting pcDNA-TRnot with the blunt-cutter PmeI and either XhoI or BamHI to create probes with overhangs on one end or the other. These fragments were then labeled with [α-32]P using the Klenow fragment (3′-5′exo-) of DNA Polymerase I (New England Biolabs) in an overhang fill in reaction. Free nucleotides were removed by gel filtration spin columns (GE Healthcare) followed by precipitation of the probes.

The binding reaction was set up in 50 µL containing a total 100 µg protein (0–100 µg mLANA-6XHis, with the remainder made up with bovine serum albumin [BSA]), 1 µg poly(dI:dC), and binding buffer (2% polyvinylethanol, 2.5% glycerol, 10 mM Tris pH 8, 0.5 mM EDTA, and 0.5 mM DTT). After a 10 minute incubation on ice, the reactions were brought to room temperature, and 50,000 cpm labeled probe was added to each reaction, and then they were incubated at room temperature for 20 minutes. DNase I (DPRF) (Worthington) was diluted into 10 mM MgCl2 and then, each reaction was exposed to 100 µL of the appropriate concentration of diluted DNase I for 30 seconds before the addition of DNase Stop buffer (8M Urea, 0.5% SDS, 5 mM EDTA) followed by two phenol extractions, three phenol-chloroform extractions, a chloroform extraction, and then ethanol precipitation. These digests were separated on an 8% denaturing acrylamide sequencing gel.

The G+A ladder was made using a modified Maxam-Gilbert protocol. 50,000 cpm probe was mixed with 2 µg salmon sperm DNA in 10 µL reaction, adding 1 µL 4% formic acid, and incubating 25 minutes at 37°C. Next, 150 µL of a 1∶10 dilution of pre-chilled piperidine was added and the reaction was incubated 30 minutes at 90°C. Then, the reaction was placed on ice, and 1 mL 1-butanol was added and vortexed. The precipitated DNA was centrifuged. The pellet was washed in 150 µL 1%SDS, then 1 mL 1-butanol was added, and the reaction which was centrifuged again. After a final wash in 0.5 mL 1-butanol, centrifugation, and removal, the pellet was dried under a vacuum and resuspended in formamide loading buffer.

### Mutagenesis of ORF73 plasmid

The plasmid pMSCV-73GFP was used to make mutants of ORF73 by subjecting it to error-prone PCR using the GeneMorph II kit (Stratagene). Conditions were set, in accordance with the manufacturer's recommendation, to create 1–2 mutations on each PCR product. The primers 73_5′ATG2 (5′-ATGCCTCCTAAAAGACGCCG-3′) and 73_3′noSTOP (5′-TGTCTGAGACCCTTGTCCCTGTT-3′) were used to amplify ORF73 from the pMSCV-73GFP (6448 ng) using the following PCR conditions: 2′95°, [30″95°, 30″55°, 60″72°]×30, 10′72°. After precipitation of the PCR product, it was used as a “megaprimer” in a site-directed mutagenesis reaction (Stratagene) on pMSCV-73GFP to create the library of mutants in the expression plasmid. Following DpnI digestion of the newly-created mutant expression, the plasmids were transformed into *E. coli*, colonies were picked, and DNA was prepared by alkaline lysis followed by silica membrane purification in 96-welll format (Zymo Research). These DNA preps were used directly for transfection and for automated DNA sequencing.

### Mutagenesis of MHV68 BAC and production of virus

The MHV68 BAC was mutated using the galK-mediated Red recombineering, as described previously for other BACs [39]. Briefly, we introduced MHV68 BAC into the galk strain of bacteria SW102. In order to create and ORF73/GalK replacement BAC, we amplified the galK gene from the plasmid pGalK using the primers galK-US-73 (5′-catgccctggcgaaggtgttgccca ggatatattctgggaatgtgatttaCCTGTTGACAATTAATCATCGGCA-3′) and galK-DS-73(5′-acccttcctgtgctaaaagttgtgactgtgtactttatctctttcagataTCAGCACTGTCCTGCT CCTT-3′); the resulting PCR product includes 50 bp of the sequence upstream and downstream of ORF73 for use in homologous recombination. The PCR product was introduced via electroporation to SW102/MHV68 cells and those recombinants were selected by using minimal media with galactose as the sole carbon source. Once ORF73/GalK replacement BAC mutants (MHV68.Δ73galK) were confirmed by restriction digest, ORF73 point mutant PCR products were made to swap out GalK. The PCR products were made by amplifying each mutant pMSCV-73GFP plasmid with the following primers, which contain the same 50 bp of homology as the primers above: 73-res-ds (5′-ACCCTTCCTGTGCTAAAAGTTGTGACTGTGTACTT TATCTCTTTCAGATAATGCCCACATCCCCAC-3′) and 73-res-us (5′-catgccctggcgaaggtgt tgcccaggatatattctgggaatgtgatttatgtctgagacccttg-3′). The mutant ORF73 PCR product was electroporated into SW102/MHV68.Δ73galK and after recombination, were selected on minimal media plates containing glycerol and 2-deoxy-D-galactose, which is toxic to bacteria that metabolize galactose. The resulting colonies were screened by colony PCR (primers 5′-ACAC AACCTCAGGCAAAAC-3′ and 5′-CCTTCAACATCAACATCTGG-3′) and then verified by restriction digest. Mutations were confirmed by PCR of ORF73 and automated DNA sequencing of the PCR product.

Resultant BACs were transfected into Vero-Cre cells. When cells reached 70% CPE, cells and supernatants were harvested by freeze/thaw lysis, and fresh Vero-Cre cells were infected with this stock to ensure excision of the BAC. One further passage in Vero-Cre cells was performed in order to expand the virus stocks.

### Virus infections and plaque assay

Virus stocks were diluted in complete media and adsorbed on cells plated the previous day. For MOI 3 infections, virus was adsorbed for one hour, the inoculum was removed, and fresh, complete media was replaced. For low MOI (0.001) infections, virus was diluted, adsorbed, and left on the cells. Virus was harvested from cells and supernatant by 3X freeze/thaw lysis. Plaque assay was performed as previously described [70].

### Immunoblot and immunoprecipitation analyses and antibodies

Immunoblots were performed as described [27]. Cells were lysed in alternative radioimmunoprecipitation assay (RIPA) buffer (150 mM NaCl, 20 mM Tris, 2 mM EDTA, 1% NP-40, 0.25% deoxycholate, 1 mM NaF, and 1 mM Na3VO4 supplemented with complete mini-EDTA-free protease inhibitors [Roche]). Antibodies used were mouse MHV68 antiserum, rabbit mLANA antiserum, mouse anti-β-actin (Sigma), as well as HRP-conjugated anti-rabbit and anti-mouse Ig antibodies(Jackson ImmunoResearch). mLANA-6XHis was detected using mouse anti-penta-His antibody (QIAGEN).

For co-immunoprecipitation experiments, lysates were prepared from 10 cm dishes of 293T cells co-transfected with p73-3XFLAG and pMSCV-73GFP (and mutants) by lysing in 1.1 mL of alt. RIPA buffer and shearing with a 20G needle. After clearing by centrifugation at 4°C, 0.1 mL was saved for whole cell lysate analysis, and the remaining 1 mL lysate was incubated overnight at 4°C with anti-FLAG (M2) agarose beads (Sigma) to pull down mLANA-3XFLAG complexes. Beads were washed according to manufacturer's instructions, and then separated on 10% SDS-PAGE gel and transferred to nitrocellulose. Blots were probed with either HRP-conjugated mouse anti-FLAG M2 (Sigma) or goat anti-GFP (Rockland Immunochemicals) and HRP-conjugated donkey anti-goat Ig (Jackson ImmunoResearch).

To generate rabbit antiserum to mLANA, the sequence of mLANA encoding amino acids 173–314 of the C-terminus was cloned into pGEX-6P-1 (GE LifeSciences), and the resulting GST fusion protein was produced in ArcticExpress BL21 cells (Agilent Technologies) then purified on Glutathione Sepharose 4B columns (GE LifeSciences).

### Mice and infections

Female C57Bl/6 mice 6 to 8 weeks of age were purchased from the Jackson Laboratory and maintained at Emory University. Mice were sterile housed and treated according to the guidelines at Emory University School of Medicine (Atlanta, GA). Mice were infected intraperitoneally with 1000 PFU of virus diluted into 0.5 mL of complete DMEM or intranasally with 1000 PFU of virus diluted into 20 µL complete DMEM. For intranasal infection, mice were sedated, and after infection allowed to recover before returning to their cages.

### Limiting dilution assays

Limiting dilution assays for frequency of latently infected cells or cells reactivating virus were performed as previously described [71], [72]. Briefly, to determine the frequency of cells harboring latent viral genomes, single-copy-sensitive nested PCR was performed on wells of serially-diluted, erythrocyte-free splenocytes. Cells were lysed by protease K digestion, and two rounds of PCR were performed per sample with twelve samples per dilution, and the products were resolved on 2% agarose gels. To measure the frequency of cells reactivating virus, splenocytes were resuspended in complete DMEM and plated in serial two-fold dilutions on mouse embryonic fibroblast (MEF) monolayers in 96-well tissue culture plates. Parallel samples of mechanically disrupted cells were plated to detect preformed infectious virus. Wells were scored for cytopathic effect 14 to 21 days post-explant. Frequencies are determined using a Poisson distribution.
